# Trends in Monoclonal Antibody Production Using Various Bioreactor Systems

**DOI:** 10.4014/jmb.1911.11066

**Published:** 2020-03-13

**Authors:** I. Jyothilekshmi, N. S. Jayaprakash

**Affiliations:** Centre for Bioseparation Technology (CBST), Vellore Institute of Technology (VIT), Vellore 632014, Tamilnadu, India

**Keywords:** Monoclonal antibodies, high density bioreactors, miniPERM or CELLine cell culture devices, hollow fiber bioreactors, cryogel bioreactor, fixed and fluidized bed bioreactors, wave bioreactor

## Abstract

Monoclonal antibodies are widely used as diagnostic reagents and for therapeutic purposes, and their demand is increasing extensively. To produce these proteins in sufficient quantities for commercial use, it is necessary to raise the output by scaling up the production processes. This review describes recent trends in high-density cell culture systems established for monoclonal antibody production that are excellent methods to scale up from the lab-scale cell culture. Among the reactors, hollow fiber bioreactors contribute to a major part of high-density cell culture as they can provide a tremendous amount of surface area in a small volume for cell growth. As an alternative to hollow fiber reactors, a novel disposable bioreactor has been developed, which consists of a polymer-based supermacroporous material, cryogel, as a matrix for cell growth. Packed bed systems and disposable wave bioreactors have also been introduced for high cell density culture. These developments in high-density cell culture systems have led to the monoclonal antibody production in an economically favourable manner and made monoclonal antibodies one of the dominant therapeutic and diagnostic proteins in biopharmaceutical industry.

## Introduction

**Fig. 1:**
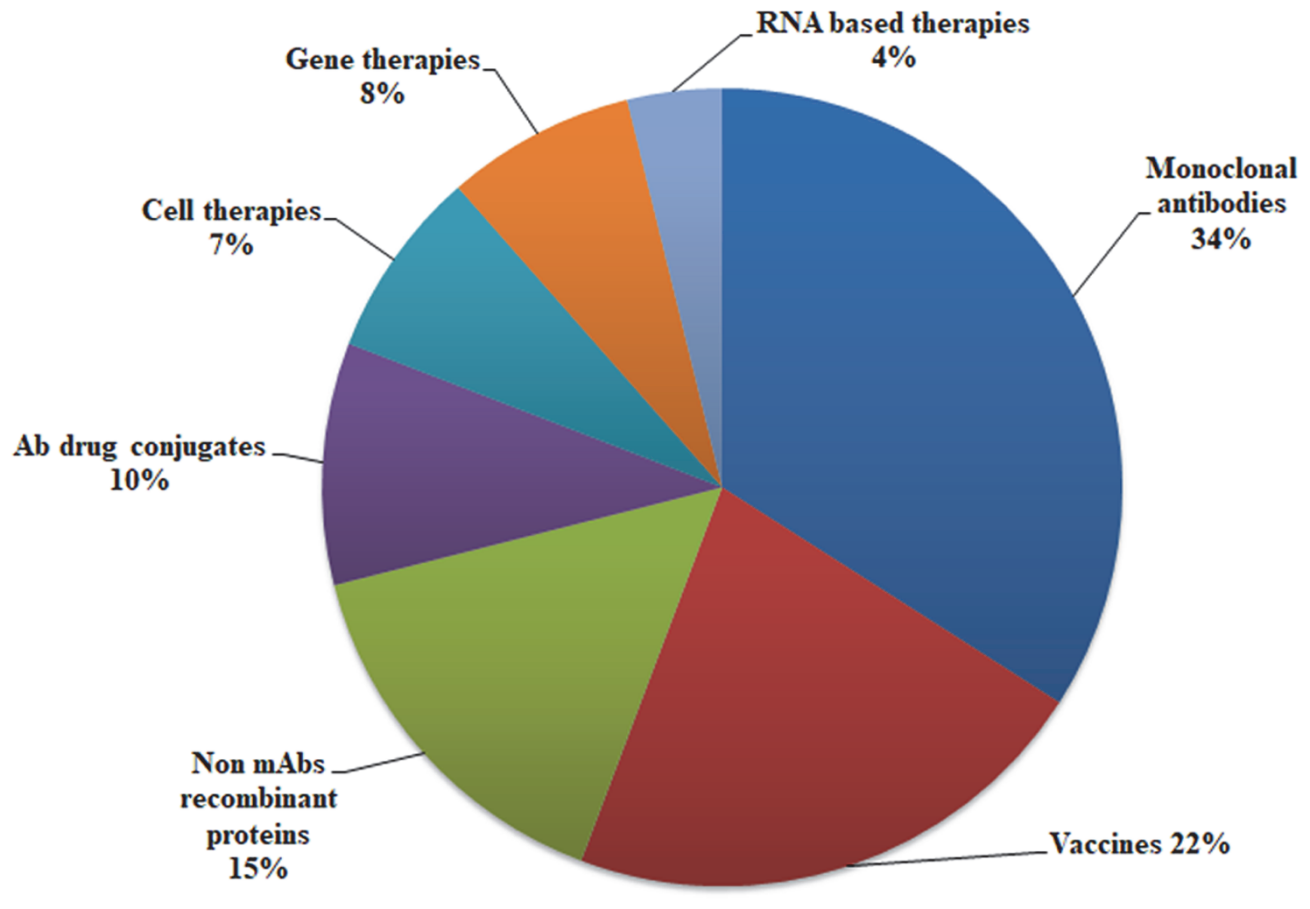
Biopharma therapeutics that are currently available according to the biopharma survey conducted by NIBRT and The medicine maker (Texere publishing) in March 2019.

The requirement of monoclonal antibodies (mAbs) as therapeutic agents and also in diagnostic applications is rising continually after the successful Nobel Prize-winning discovery of hybridoma technology by Georges Kohler and Cesar Milstein in 1975. The production of safe, efficacious and affordable monoclonal antibodies in surplus quantities warrants advanced process strategies to overcome the disadvantages of conventional methods. The commercial development of monoclonal antibody for therapeutic development was established inthe early 1980s and the first approved monoclonal antibody was OKT3 for the prevention of kidney transplant rejection in 1986 [1].Attempts were also made to improve the efficiency of antibodies either by the production of chimeric monoclonal antibodies which contain murine variable regions and human Fc IgG component [2] or by producing fully human monoclonal antibodies [3]. According to Biopharma trend survey March 2019, the major portion among biopharma products in the product pipeline is monoclonal antibodies (Fig. 1) [4]. There are 31 new mAbs and 10 biosimilars that had been introduced to the market since 2013, which made the global market in a total of 51 mAbs and 11 biosimilars at the end of 2017 [5]. Although the global market of mAbs is progressing well, there were some crucial challenges concerning mAb manufacturing such as the process robustness, product reproducibility, product yield, and characterization. The failure in such challenges led to the rejection of some drugs from the approved list, for example, withdrawal of the medicine Zynbryta for multiple sclerosis in March 2018 because of the symptoms of brain inflammation. It has been reported that, among the approved mAbs, the highest number of mAbs targets cancer, which is 15mAbs and 12 mAbs targets hematology, followed by dermatology (9 mAbs), rheumatology (8 mAbs) and so on [5]. In 2018, twelve therapeutic antibodies which treat a variety of disease were granted approval in European Union (EU) or US, which include three for migraine prevention, two for cancer and also HIV infection. It has been reported that there are four monoclonal antibodies undergoing regulatory review which is for different therapeutic areas for triple-negative breast cancer, paroxysmal nocturnal hemoglobinuria, plaque psoriasis, and osteoporosis. It was found that almost 33 therapeutic antibodies for cancer are in the last stage pipeline of clinical studies for chronic lympholytic leukemia, diffuse large cell lymphoma, multiple myeloma, breast cancer, melanoma, bladder cancer and so on [6]. The rapid increase in the approval of mAbs regularly for therapeutic use puts up a need to produce sufficient quantities of mAbs. Although the development and production of mAbs occur under strict guidelines of regulatory authorities, the use of advanced technologies and ever-increasing familiarity of mAbs will contribute their dominance in the biopharmaceutical industries.

## Track to the Emergence of High-Density Cell Culture Systems

Commercially, mAbs are produced in two different ways *i.e.* by in vivo and in vitro technology. Nowadays, the in vivo method has diminished due to animal ethical concerns. The in vitro technology, involving small-scale suspension cell culture production of mAb, utilizes devices such as T-flask, Triple flask, Cellstack, Hyper flask, spinner flasks, roller bottles and shake-flasks [7]. In these conventional systems, the monoclonal antibody concentration is limited between 10 and 100 μg/ml, because of their low cell density. On contrary to the above mentioned conventional methods, perfusion bioreactor systems have been developed. The perfusion bioreactors are capable of providing long term culture stability for cells by continuously feeding with fresh media and removing spent media thereby eliminating the metabolic waste products which hinder cell growth. Therefore, to produce mAbs in higher quantities, many different types of perfusion based high cell density bioreactor systems are available. The Table 1 gives some of the examples of mAbs, source cell line and their cultivation methods. The operating conditions of different bioreactors for the upscaling of specific monoclonal antibody production have been studied. The roller bottles, stirred tank and disposable bioreactors have been operated with both serum and serum-free media. It was found that the cells were not able to adapt to serum-free media in roller bottles and stirred tank bioreactors [12]. The highest monoclonal antibody titre was observed in a disposable static membrane bioreactor and the peak productivity was found in a stirred tank bioreactor operated in semicontinuous mode with overlay aeration [12]. Serum free medium has also been successfully used in high cell density bioreactors such as hollow fiber systems [18], packed bed reactors [19] and wave bioreactors [20] for the cultivation of mammalian cells. There is a considerable progress in cell culture techniques using serum free media, that supports the growth of hybridoma cells [21] and CHO cell lines [22, 23] for therapeutic protein production. To reduce the cost of production and to increase the output in therapeutic protein production, high-density culture (≤10^7^ cells/ml) systems have been introduced. Eventually, the production of monoclonal antibodies with high cell density and good cell viability has become the need of the hour and this review focuses on popularized high cell density culture systems for monoclonal antibody production.

According to the property of cell lines, they can be either cultured in suspension or adherent culture. Accordingly, a copious amount of work has been done for the suspension culture of cell lines in stirred tank bioreactor for monoclonal antibody production. The perfusion mode culture in the stirred tank reactor with the ceramic membrane as a cell retention device was used for the production of IgG 2a mAb. The maximum cell count was reported to be 3.5×10^7^ cells/ml and the cell viability was found to be 95% such that when this system is used with micro-carriers or porous microspheres or perfusion technique, the cell density will reach up to 10^7^ cells/ml [24]. Another study reported that stirred tank bioreactors are widely used for the production of mAbs in industrial large-scale for cell lines like CHO, hybridoma, and NS0. The perfusion mode of culture with stirred tank bioreactors using spin filters significantly resulted in high volumetric production of mAb which is specific for endofloxacin with a yield of 61.4mg/day and achieved the cell viability of 1.57 × 10^6^ cells/ml within 5 days [14]. Some cell lines are incapable of growing in suspension; for such cell lines, the high cell density, low volumetric systems are available which means that cells can achieve high cell density in a small volume.

Although the stirred tank bioreactors could be considered as high cell density culture bioreactors, the hybridoma cell lines could not bear the shear stress by suspension media agitation. Therefore, it is important to improve the techniques for immobilizing cells to porous beads. Cells are usually immobilized by adsorption or entrapment in high cell density culture systems. The size, porosity and surface characteristics of the matrix determine the efficacy of cell adsorption. The matrices could be natural/synthetic or may require some chemical modifications for the adsorption of cells. Packed bed and polymer-based bioreactors systems have been typically used for the immobilization of cells for cultivation. A high cell density culture system renders compact bioreactors with high volumetric production, although these systems are difficult to operate [25].

## High Cell Density Culture Systems

### Hollow Fiber Bioreactor

The first description of the hollow fiber bioreactor (HFBR) was given by Knazek *et al*. [26], which showed that mammalian cells were able to attach to the hollow fiber and grow in vitro. This approach has many advantages, it can attain the cell density similar to the growth of solid tissues in vivo and cells could be maintained in a more physiological state concerning nutrient supply, waste removal, and pH control.

**Fig. 2:**
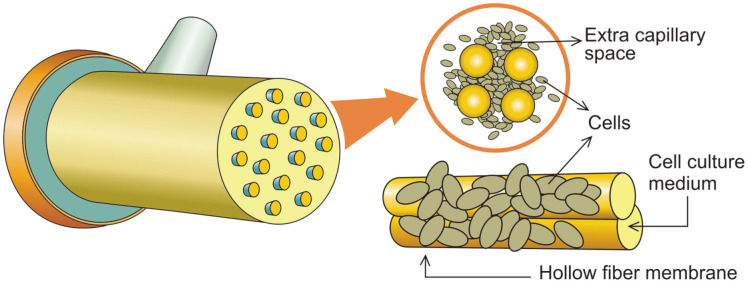
Representation of hollow fiber bioreactor which consists of a bundle of fibres and media circulated within capillaries while cells are grown in the extra capillary space.

The hollow fiber perfusion bioreactor is a continuous perfusion culture system, containing a set of thousands of semipermeable hollow fibres of 200 μm diameter in a parallel array within a cartridge with inlet and outlet ports (Fig. 2). The cells are placed on the outside of the fiber in the extra capillary space where they can attach and grow. The cell culture medium is continuously circulated through the interior of the fibres to provide nutrients and for oxygenation and hollow fibres allow nutrients and metabolic waste products to diffuse both the ways across the fiber walls. A wide range of materials is used for the hollow fibres like polysulfone and cellulose derivatives, which are hydrophilic and have high percent porosity. The molecular weight cut-off begins at 5 kDa and goes up to virtually any desired limit. Hollow fibers provide a high surface to volume ratio of 200 cm^2^/ml [27] and allow a large number of cells to attach in a small volume. Due to the avoidance of nutrient inadequacy and accumulation of toxic metabolites, cells can achieve a high cell density of 10^9^ cells/ml. Antibodies can be produced from a single cartridge for more than six months in continuous culture mode. In a study, the production of antibody fragments in hollow fiber bioreactors resulted in the antibody harvest around 137 to 307 mg in the crude extract. The further purification protocol, which includes hydroxy-appetite and ion-exchange chromatography, showed a good recovery of 50 to 150 mg of purified antibody fragments [33]. In hollow fiber bioreactors, the cells are also allowed to culture in reduced serum or serum-free medium which facilitates the product for the convenient purification [28-30]. Accordingly, with serum-free medium it was possible to run the hollow fiber bioreactor for forty three days. The mAb production was 11 mg/ day and the maximum amount of IgG obtained was about 2.4 mg/ml [31].

As the cells grow in high densities, the nutrients must be transferred across the layer of cells, so the mass transfer that occurs due to diffusion is limited to several cells. Subsequently, many efforts have been made to modify the design of HFBR, because of their popularity in high-density culture systems.The development of the axial flow HFBR system was one of the breakthroughs in the HFBR development. To overcome the limitations of mass convection transport by the previous system, a new approach called tricentric HFBR has been developed. As compared to the conventional HFBR, tricentric HFBR offers a third compartment that is meant for immobilized cells and the other two compartments, namely intra and extra capillary compartments are dedicated for medium flow.As an alternative to conventional HFBR, a new system called micro HFBR has also been developed. It consists of fibers that are implanted inside silicon tubing where the cells are seeded inside the fibers and the extra capillary space between the fibers and silicon tubing is satisfactory for medium supply.

Another HFBR called Fiber Cell systems have been introduced, which consist of hydrophilic polysulfone fiber for good cell culture performance with MWCO of 5 kDa and 20 kDa [32].One unique feature of the Fiber Cell system is that the fibers are cast with little waves, which result in even distribution of the fiber bundles within the cartridge shell. These waves push against one another to produce uniform fiber bundles, by eliminating channelling effect by the fiber spacing. Hollow fiber bioreactors are permissible for both evaluation of scale-up and also for non-affinity based purification protocol.

Hollow fiber bioreactors have vast amount of applications in industrial aspect in the near future, as those are highly efficient and compact. The HFBs are highly economical for virus cultivation and also for toxicology studies in vitro, which helps to model the availability of drugs and dosage. Studies are on-going with hollow fibres in the field of tissue engineering to utilise their property of biocompatibility with the cells. The surface property of hollow fibers facilitates the stem cell expansion, 3D culture and engineering of soft tissues.

The introduction of dialysis membrane-based bioreactors, namely MiniPERM and CELLine 1000 had been also used for high-density cell culture. The MiniPERM bioreactor consists of two chambers separated by a dialysis membrane. These two chambers are the production module and supply module for antibody production and medium supply respectively. The outside of the production module is made up of silicon tubing which helps in the exchange of gas and nutrients and low molecular weight compounds pass across the dialysis membrane by diffusion; whereas, in CELLine system, the operation of the system is simple like a tissue culture flask. These systems consist of a dialysis membrane of 10 kDa, which separates the cell compartment from medium reservoir. The silicon membranes on the base of the cell compartment facilitate gas exchange. Both the dialysis membrane-based bioreactor systems reach the cell density of about 10^7^ to 10^8^ cells/ml, which is about two times higher than the one obtained from conventional cell culture methods.

### Fixed and Fluidized Bed Bioreactors

**Fig. 3:**
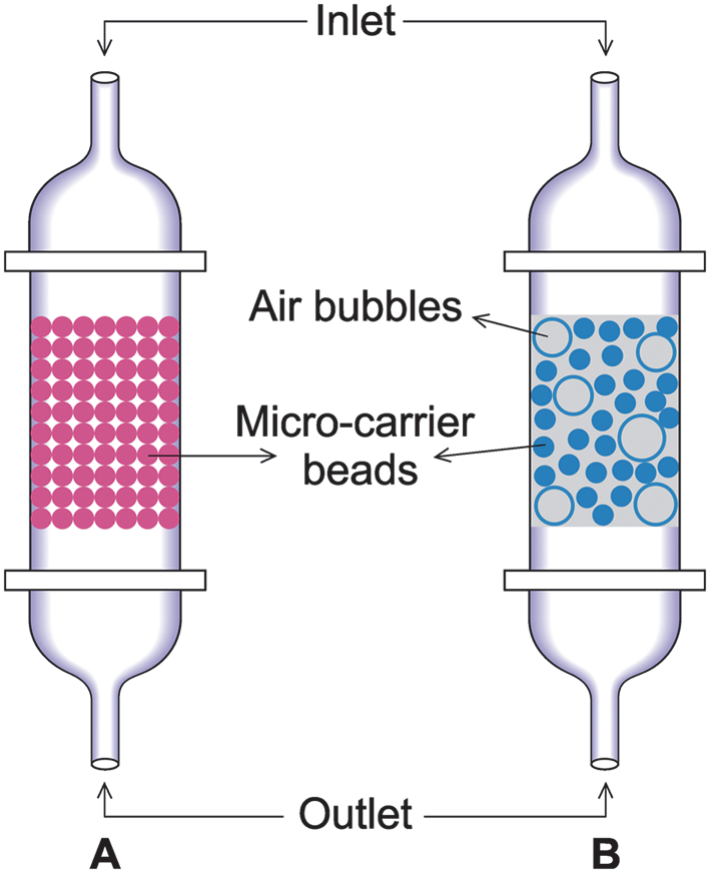
Schematic representation of fixed and fluidized bed reactor systems with cells immobilized over micro-carrier beads (A) Fixed bed reactor (B) Fluidized bed reactor.

Earlier studies with fixed and fluidized bed reactors focussed on the production of therapeutic proteins as an alternative to hollow fiber bioreactors. These systems have been successfully used for many cell lines for the viral construct in gene therapy and also the cultivation of cell lines such as hybridoma, CHO, VERO, etc. In the case of fixed and fluidized bed reactors, the systems consist of a column filled or packed with microcarrier beads for the support where the cells get immobilized (Fig. 3). In detail, these microcarrier beads are retained in a cylindrical vessel, through which the media circulates. In fixed-bed systems, the microcarriers are packed in a column whereas, in fluidized systems, the microcarrier porous beads float in a column. The column is continuously perfused with media from the reservoir. They have been successfully applied for the long term production of monoclonal antibodies. The most commonly used porous microcarriers in packed bed systems are Fibra-Cel and SIRAN. Fibra-Cel microcarrier beads can achieve cell density and productivity ten times higher as compared to conventional methods. It has been reported that these types of microcarrier systems can contain cells up to the density of 10^8^ cells/ml even in serum-free media [34]. Cytoline (GE healthcare) is the microcarrier most commonly used in fluidized bioreactor systems which achieves the cell density of 2 × 10^8^ cells/ml. Cytopilot Mini is a fluidized bioreactor that uses the cytoline as a microcarrier and capable of producing 50 mg/l of monoclonal antibodies in a running culture for 60 days. The microbeads packed in a vessel with continuous media supply could achieve high cell density, high metabolic activity, and homogeneous cell distribution. Recent studies which have carried out with fluidised bed systems showed their importance in present and future of bioprocessing technology. Fluidised bed bioreactors have improved in a way to minimize the void volume in the microcarrier beads. In this process, microcapsule suspension fluidised system has been developed, which resulted in high viability percentage and there by improved the productivity [35]. In another study, centrifugal bioreactors have been developed similar to fluidized bed bioreactor. In centrifugal bioreactor systems, the cells were immobilized on to the microcarrier beads by centrifugal force, which achieved high cell density and good cell viability also. It is reported that the cell density increases up to 1.7 times approximately when the centrifugal force is doubled [36].These studies reveal that, the fixed and fluidised bed bioreactor systems become one of the pioneers in bioprocessing in the future.

### Wave Bioreactor

**Fig. 4:**
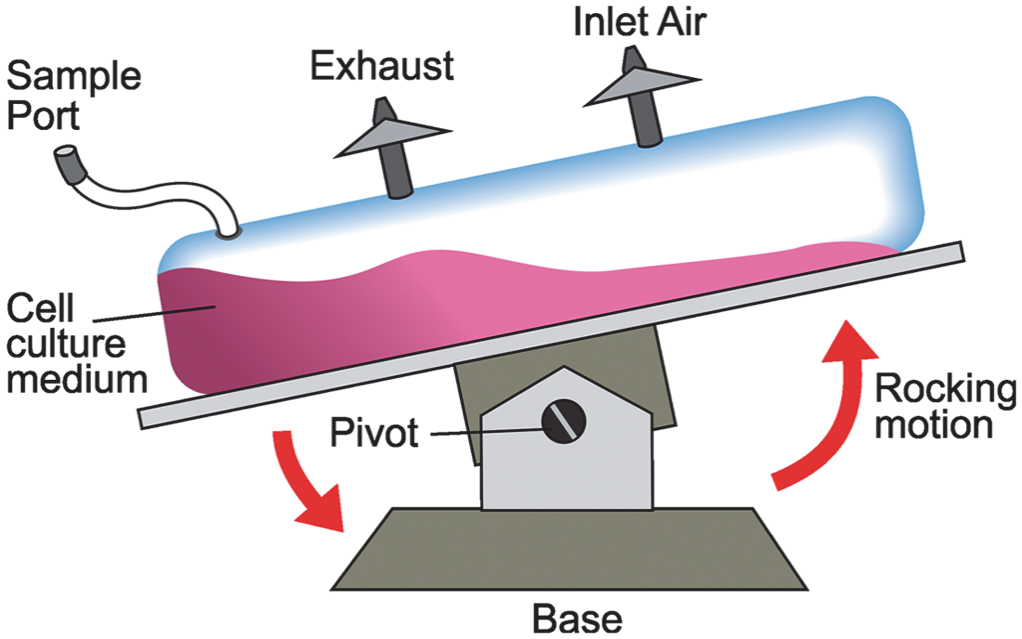
Schematic representation of wave bioreactor, tilting along a pivot axis causing the movement of cell culture medium with cells by rocking motion.

These bioreactors are efficient, cost-effective systems in bioprocessing and this reactor has been tested for cell lines like hybridoma cells, CHO cells, NSO cells and also insect cells. Wave bioreactors are a kind of disposable bioreactors, designed by Singh for the cultivation of mammalian cells [37]. The structure of the wave bioreactor consists of a disposable chamber called cell bag, which contains the cell culture medium, cells and also a port of air circulation (Fig. 4). The mixing and agitation are enhanced by the rocking motion of the chamber backward and forward, which leads to efficient mass transfer. Hence, the resulting ambient environment for cell growth can achieve a cell density over 1 × 10^8^ cells/ml and this system can be scaled up to 1,000 L. In this kind of wave bioreactor, the cells are inoculated into two-in-one cell bags, where cell growth and antibody production can be monitored separately for batch and perfusion cultures. Thus, maximum cell viability in perfusion mode was found to be 1.04 × 10^4^ cells/ml and antibody production was 1,437 mg/l·day. Recent studies carried out with the wave bioreactors showed that, these systems are apt for virus cultivation [38], insect cell cultivation [39], expansion of embryonic cells etc. Eventually, the wave bioreactors will have a future outlook for the production of high value proteins from virus, which are difficult for upstream processing.

### Cryogel Bioreactors

**Fig. 5:**
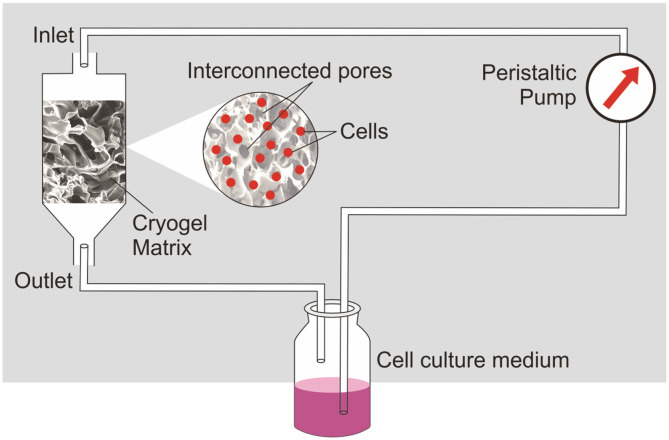
Diagrammatic representation of cryogel bioreactor, where cells are entrapped into the pores of the cryogel matrix and replenished by the continuous mode of the medium supply.

The vital necessity of developing economically favourable bioreactors in both lab-scale and industrial level leads to the debate of disposable bioreactor systems. Cryogel bioreactor is a disposable, esteemed, high cell density perfusion bioreactor, which gives a long productive lifetime for cells. It has been described for long term continuous production of therapeutic proteins [40-42]. The bioreactor consists of a polymeric cryogel as a matrix for the cultivation of cells, medium reservoir and peristaltic pump (Fig. 5). Cryogels are three-dimensional polymeric scaffolds formed at subzero temperature by polymerization of monomers or by polymeric precursor by the phenomenon of cryogelation, which consists of an interconnected network of macropores. Cryogelation technique has an advantage that the cryogels can be made in different sizes and shapes like disc, sheets or monoliths with varying dimensions. The polymer-based cryogel has several advantages over other kinds of gels *i.e.*, simple approach by which they can be synthesized,use of aqueous solvent for their synthesis and unique combination of high porosity with adequate mechanical strength and osmotic stability. The size of these macropores varies from few micrometers to 100 μm,which allows the unhindered convectional mass transfer. This makes cryogels an ultimate support for cell immobilization and proliferation. The hybridoma cell lines are adsorbed to the inner pore walls of cryogel matrices which are covalently immobilized with gelatin. The coating with gelatin enhances the adherence of cells to the matrices. The cells are situated in such a microenvironment which makes sure that there is virtually no barrier that arises for easy diffusion of substrates and metabolites [43, 44]. As observed in the literature, it has been reported that a bioreactor with polyacrylamide based cryogel was run in continuous mode for 55 days and obtained monoclonal antibody productivity of 130 μg/ml on the 36^th^ day [42].

The future advances of cryogel matrices are relayed on their property of being biocompatible with any cell line. Hence, cell based therapies require enormous number of cells with cell density ranging from 10^8^ to 10^10^. Cryogel matrices are the efficient platform to grow large quantity of cells in order to avoid the restriction of space required for the growth of cells because of their high surface area. In addition to that, the studies regarding microfluidic bioreactors with cryogels is also on-going, that will make the future brighter for the 3D modelling of cells, followed by ‘organ on a chip’ technology. Therefore, the establishment of such low investment platforms will have significant benefits on understanding disease development and drug modelling. Apart from the cell culture applications, cryogel bioreactors have also been used for industrial production of biomolecules. In a study, cryogels were immobilised with α-amylase for starch hydrolysis for the industrial production of maltose [45]. Those studies are the forthcoming indication for the acceptance of cryogel bioreactors in industrial production of various biomolecules.

## Recent Developments in High Cell Density Culture Systems

To improve the performance of high cell density bioreactors, a considerable amount of work has been done by utilizing new technologies. Some of the bioreactors which have been working at high cell density are listed in Table 2. In case of productivity, major improvements have been done in two different aspects. One of them is to improve the cellular productivity by modification in the growth environment [52, 53] and the other method is to increase the volumetric productivity where the cell density is relevant [54, 55]. Significant efforts have been made with some studies providing insights into the use of bioinformatics tools for the upmarket production of monoclonal antibodies. One of them is the designing of 3D nylon beads for cell immobilization studies. Thus, this technique could utilize 3D nylon beads of different bead sizes of 15 to 30 nm with a varying pore size (0.6, 1, and 1.5 mm). The use of such 3D beads provided easiness in handling and saved the time and resources [56]. The computational analysis was exploited for the modelling of a high-performance agent band to create changes in different variants of cell culture to simulate mammalian cell culture bioreactor with the notified increase in cell viability [57].

Proteomics had also provided some new insights into bioreactors processing and enhanced the production of therapeutic proteins. It is important to identify the parameters which affect the production of a particular protein. Further studies revealed that the monitoring of the parameters with microarray-based MALDI-TOF had achieved the production of monoclonal antibodies with high quality at the end, which could guide further processing in biopharma [58].

Many factors affect the proliferation of high cell density cultures including pH, temperature, dissolved oxygen, nutrient consumption, metabolic accumulation, serum growth factor, extracellular matrix, etc. It was possible to improve the robustness, high cell viability and also high productivity through high cell density by a small change in the pH in perfusion culture [10]. In another study, to boost up the monoclonal antibody production with CHO cell lines, the pH of cell culture media was optimized for pH control strategy using pH-dependent dynamic model. This process of pH shift towards the proper range suitable for the CHO cell lines resulted in a 40% increase in the monoclonal antibody production [59]. Likewise, cell culture media contributes a major role in cell viability and product quality. The optimization of cell culture media through the supplementation of various amino acids showed improved production of monoclonal antibodies. It has been shown that the amino acid input to the cell media leads to the high cell density of CHO cell lines and produced good metabolic profiles [60].

Apart from the modification with the cell culture parameters, the existing high cell density bioreactors have been renovated in many aspects from the past ten years. Eventually, a new concept of bioreactor named cell tank has been introduced, which is a disposable mode of bioreactor for perfusion mode of culture. It can be used for both adherent and non-adherent cell lines at high cell density and consists of a non-woven polyester matrix caged in a cassette and immersed in a reservoir. Cells are entrapped in the matrix and the media circulates through it [61]. A novel bioreactor system has also developed recently which mimic the scale down perfusion culture. This system studied the cell retention by sedimentation, and then cell culture supernatant was removed and replenished with fresh media. This approach could obtain good cell densityand also could reduce the cell culture media requirements up to 80 fold [62].

Hollow fiber bioreactors are one of the most influencing systems in high cell density culture. Recent advancements in the field of hollow fiber bioreactors aided in improved production of therapeutics. Microfiltration based hollow fiber bioreactors were developed which consist of two hollow fibers inserted in a vessel, which could able to remove low molecular weight inhibiting metabolites without removing the cells [63]. A crossed hollow fibre membrane bioreactor consists of two types of fibres with a different molecular weight cut off (MWCO) which also possesses entirely different physicochemical properties [64]. Hollow fibres have the disadvantage of membrane fouling, which further reduces the life of the same. In order to reduce this issue, studies have been carried out to develop hollow fibres with large pore size. The development of such hollow fiber systems with large pore size could offer good cell retention while allowing complete passage of product with achievement of high cell density [65].

The hollow fiber bioreactors and membrane bioreactors have some common disadvantages like the risk of contamination, clogging of cells in the membrane; difficulty in monitoring, which led to the development of matrices for the immobilization of cells. Among the high cell density culture systems where the matrices have been used for cell immobilization, the contribution of cryogel bioreactors was boundless. In one study, poly-D-lysine was used for cell adhesion in microfluidic chips for the hybridoma cell lines, which provided good cell viability and long term culture stability [66]. Gelatin has been also used for coating over the cryogel inner walls for cell adhesion. Cryogel bioreactors are well admired in many studies for the production of therapeutic proteins including monoclonal antibodies. The major advantage was that cryogels matrices consisting of different polymeric blends could be prepared. Therefore, cryogel matrices could be prepared by a combination of chemically different monomers which yielded the cryogel’s surfaces either charged or uncharged. The ease of preparing cryogels at different shapes also made the drift towards cryogel bioreactors for high cell density and long term culture stability [67]. Eventually, cryogel bioreactors have been used for the upscaling of many mAbs and also for other purposes like wastewater treatment and *in vitro* model for cancer metastasis studies [68]. The ease and handling of cryogel matrices broaden their applications in different fields like bioseparation, tissue engineering, etc. Especially in cell culture, cryogel matrices were used as 3D support for cells to study their growth parameters especially from cancer cell lines [69]and bone cells. Subsequently, in a recent research study, cryogels were used for the storage and transportation of mammalian cells. The cell viability was found to be approximately 1.5 times higher in comparison with suspension culture and the duration of cryopreservation in cryogel matrices doesn’t affect the cell viability [70].

Many attempts have also been made in the case of wave bioreactors. In particular, the perfusion culture using a wave bioreactor was demonstrated for the production of human monoclonal antibody from *Drosophila* Schneider 2 cells [13]. The perfusion mode operation of wave bioreactor using microfiltration or ultrafiltration by altering tangential flow (ATF) or tangential flow filtration (TFF) leads to the growth of CHO cell lines up to 10^8^ cells/ml after twelve days and mAb production was found to be six times higher [48]. The introduction of low-intensity pulsed ultrasound to wave bags increased the production of mAb. It was found that five minutes of ultrasound treatment increases the antibody titre up to 25% [11]. In another example, a new technology-based 2D rocking bioreactor has been established and utilized for recombinant protein production. It is a CELL-tainer bioreactor system and is an alternative to the stirred tank reactors for the bioprocessing centered on microbial platforms [71].

In the biopharma industry, to speed up the ever-present drive to increase the product yield, it is necessary to improve the existing systems for both manufacturing and upstream processing. Thus, significant efforts have been made for upgrading the processes for the monoclonal antibody production and also to increase the efficiency of the existing processes. High-density cell culture systems provide high volumetric productivity and that bioreactor system seems to be perfect for both anchorage-dependent and suspension cultures. The entry of porous matrix for cell immobilization leads to a potential peak in the graph of therapeutic protein production. Cryogel based bioreactors are one among the disposable bioreactors, which provide significantly large surface area for cell immobilization thereby elicit higher monoclonal antibody production in lab-scale and commercial production. Also, the productivity of cryogel based bioreactors is equally comparable with that of hollow fiber bioreactors.

## Figures and Tables

**Table 1:** Monoclonal antibodies, source cell line and cultivation method.

Sl. No	Antibody	Host cell line	Culture system	Ref
1	RF-HBs-1(Anti HBs Ag mAb)	Hybridoma	Perfusion	[8]
2	CB72.3 Chimeric IgG4	GS-CHO 46	Fed Batch /Perfusion	[9]
3	Alemtuzumab(Anti CD 52 mAb)	CHO	Perfusion	[10]
4	Anti IL-8 mAb	CHO	Shake flask /wave bag	[11]
5	Anti-Salmonella Enteritidis O-Ag mAb	Hybridoma	Roller bottles/Stirred tank/Disposable bioreactor	[12]
6	100F4 mAb (specific to HA protein of H1N1 influenza)	Drosophila Schneider 2	Perfusion	[13]
7	Anti enrofloxacin IgG1 mAb	Hybridoma	Batch/Fed Batch/Perfusion	[14]
8	CRL-1606 (Anti fibronection IgG mAb)	Hybridoma	Batch/Fed Batch	[15]
9	Anti digitoxin IgG	Hybridoma	Batch/perfusion	[16]
10	Anti CD22 IgG_2_a	SP _2/0_	Batch/Fed Batch/Perfusion	[17]

**Table 2:** High cell density bioreactor systems for monoclonal antibody production.

Type of bioreactor	Cell density (Cells/mL)	Period of run (Days)	Advantages	Disadvantages	Ref.
Hollow fiber bioreactor	1-2x10^8^	30	• Reduces serum requirement • Increase in secretary product concentration(10X to 100X) • Cells grow post fluently for months	• Membrane fouling • Fiber breakage • Difficult to monitor	[46] [47]
Wave bioreactor	0.3-1.5x10^8^	27	• Flexibility • Minimum time and cost	• Non customisable • Restricted scale up	[48]
• Fixed bed reactor • Fluidised bed reactor	5x10^7^-1x10^8^ 5X10^8^	24 24	• Easy to build • Low cost of construction,operation and maintenance	• Increased vessel size and pressure drop • Erosion of internal components	[49] [50]
Cryogel bioreactor	1x10^7^-1x10^8^	55-60	• High biocompatibility • Cells are resistant to degradation • Non-toxic	• Non reusable	[51]
